# OTUB2 promotes proliferation and metastasis of triple-negative breast cancer by deubiquitinating TRAF6

**DOI:** 10.32604/or.2025.062767

**Published:** 2025-04-18

**Authors:** YU QIU, RUIHAN LIU, SHANSHAN HUANG, QIAOTING CAI, YI XIE, ZHITING HE, WEIGE TAN, XINHUA XIE

**Affiliations:** 1 State Key Laboratory of Oncology in South China, Guangdong Provincial Clinical Research Center for Cancer, Sun Yat-Sen University Cancer Center, Guangzhou, 510030, China; 2 Department of Breast Surgery, The First Affiliated Hospital, Guangzhou Medical University, Guangzhou, 510030, China

**Keywords:** OTUB2, Tumor necrosis factor receptor-associated factor 6 (TRAF6), Triple-Negative Breast Cancer (TNBC), Deubiquitination

## Abstract

**Objectives:**

Deubiquitinase OTUB2 plays a critical role in the progression of various tumors. However, its specific role in triple-negative breast cancer (TNBC) remains unclear. This study aims to elucidate the biological function of OTUB2 in TNBC and uncover the underlying mechanisms.

**Methods:**

First, we found that the expression of *OTUB2* was upregulated in TNBC by bioinformatics analysis, we then validated its expression in TNBC tissues and cells using immunohistochemistry (IHC) and qPCR and plotted the survival curves by Kaplan-Meier method. Gene set enrichment analysis (GSEA) suggested that OTUB2 may be involved in tumor proliferation and metastasis. Further functional assays, including Cell Counting Kit-8 (CCK-8), colony formation, Transwell, and wound healing assays, were performed to assess the effects of OTUB2 overexpression and knockdown on TNBC cell proliferation and migration. Additionally, UbiBrowser 2.0 was used to identify OTUB2 substrate proteins and western blotting was conducted to clarify the molecular mechanisms involved.

**Results:**

Our results demonstrated that OTUB2 expression was elevated in TNBC and associated with poor prognosis. Overexpression of OTUB2 enhanced the proliferation and migration of TNBC cells, while its knockdown inhibited these processes. Moreover, OTUB2 stabilized tumor necrosis factor receptor-associated factor 6 (TRAF6) by deubiquitinating it, leading to activation of the protein kinase B (AKT) pathway.

**Conclusions:**

OTUB2 exerts its promoting effects on the progression of TNBC by activating the TRAF6/AKT pathway.

## Introduction

Breast cancer is one of the most prevalent malignant tumors globally and ranks as the second leading cause of cancer-related mortality among women [1]. Triple-negative breast cancer (TNBC) is characterized by the absence of estrogen receptor (ER), progesterone receptor (PR), and human epidermal growth factor receptor-2 (HER2) expression, which results in a lack of effective therapeutic targets and classifies it as the subtype with the poorest prognosis within breast cancer [2]. Although TNBC accounts for only 15%–20% of all breast cancer cases, it accounts for over 80% of breast cancer fatalities [3,4]. Traditional chemotherapy agents, including paclitaxel, anthracyclines, and platinum-based drugs, remain foundational to first-line treatment for patients diagnosed with triple-negative breast cancer. However, the high likelihood of recurrence and metastasis significantly undermines clinical outcomes [5,6]. Consequently, there is an urgent need to identify novel therapeutic targets and develop innovative treatment strategies aimed at improving survival rates for patients suffering from TNBC.

As a vital post-translational modification in eukaryotic cells, protein ubiquitination and deubiquitination play pivotal roles in regulating numerous physiological and pathological processes, including cellular growth and differentiation as well as tumor progression [7]. Deubiquitinases (DUBs) are enzymes that reverse ubiquitination by removing ubiquitin from specific target substrates, thereby stabilizing their expression. Recent studies have demonstrated that deubiquitinases significantly influence cancer progression and have emerged as promising novel targets for anti-cancer therapies [8]. For instance, USP36 deubiquitinates ALKBH5 to facilitate the progression of glioblastoma [9], and JOSD2 stabilizes YAP/TAZ to enhance the advancement of cholangiocarcinoma [8].

The human genome encodes more than 100 deubiquitinases, which are mainly classified into seven families, including the ubiquitin-specific proteases (USPs), the ubiquitin carboxyl-terminal hydrolases (UCHs), and the otubain/ovarian tumor-domain containing proteins (OTUs) [10]. In recent years, OTUB2, a member of the OTUs family, has been reported to be involved in various tumorigenic processes, including the tumorigenesis of non-small cell lung cancer through the AKT/mTOR signaling pathway [11]. Nevertheless, the pathophysiological function of OTUB2 in triple-negative breast cancer remains indistinct.

Here, we identified the upregulation of the deubiquitinase OTUB2 in TNBC through differential analysis, both *in vivo* and *in vitro* experiments showed that OTUB2 significantly promotes TNBC cell proliferation and migration.

## Materials and Methods

### Bioinformatics analysis

The RNA-seq gene expression and clinical information of breast cancer samples were obtained from the TCGA-BRCA dataset via UCSC Xena (https://xenabrowser.net/) (accessed on 02 March 2025) [12]. Based on receptor status determined by immunohistochemistry, 113 normal breast tissue samples, and 121 TNBC samples were selected for differential expression analysis. The analysis was conducted using the R package “limma” (version 3.62.1) in R software (version 4.2.2) [13]. The list of deubiquitinases was obtained from the DUBase database (https://ehubio.ehu.eus/dubase/) (accessed on 02 March 2025) and provided in the supplementary materials. Deubiquitinases with significant differential expression were identified based on the criteria of |log_2_FC| > 0.585 and adj *p*-value < 0.05. Additionally, the GSE21653 dataset was analyzed for differential expression using the GEO2R online analysis tool (https://www.ncbi.nlm.nih.gov/geo/info/geo2r.html) (accessed on 02 March 2025) available on the GEO platform (https://www.ncbi.nlm.nih.gov/geo/) (accessed on 02 March 2025) [14]. Sangerbox (http://www.sangerbox.com/) (accessed on 02 March 2025) was utilized as a comprehensive bioinformatics analysis platform [15]. The pan-cancer dataset (TCGA TARGET GTEx, PANCAN, N = 19,131, G = 60,499) was downloaded from UCSC Xena (https://xenabrowser.net/) (accessed on 02 March 2025), which provides uniformly processed and standardized expression data. The expression levels of ENSG00000089723 (OTUB2) were extracted from this dataset and analyzed across different cancer types. The TIMER (Tumor Immune Estimation Resource, http://timer.cistrome.org/) (accessed on 02 March 2025) and GEPIA2 (http://gepia2.cancer-pku.cn) were utilized to further validate the differential expression of the gene across pan-cancer datasets. [16,17]. In this study, box plots generated by GEPIA2 and UALCAN (http://ualcan.path.uab.edu) (accessed on 02 March 2025) were used to visualize *OTUB2* expression in TNBC [18]. Survival analysis was conducted using the Kaplan-Meier plotter (https://kmplot.com/analysis/) (accessed on 02 March 2025) database to evaluate the effect of *OTUB2* on recurrence-free survival (RFS) in breast cancer and triple-negative breast cancer. The protein-protein interaction (PPI) network was constructed using the STRING database (version 12.0, https://www.string-db.org) (accessed on 02 March 2025) with a confidence score threshold of >0.7 [19]. The resulting work was then downloaded and further analyzed in Cytoscape software (version 3.6.0). UbiBrowser (http://ubibrowser.ncpsb.org/ubibrowser/) (accessed on 02 March 2025) is a web-based application that utilizes a naïve Bayesian computational framework to reliably predict interactions between human E3 ubiquitin ligase and their corresponding substrates [20].

### Gene set enrichment analysis (GSEA)

The gene expression profiles of 121 TNBC specimens from the TCGA-BRCA dataset were analyzed to explore gene expression differences based on *OTUB2* expression levels. Gene set enrichment analysis (GSEA) was performed using GSEA software (version 4.0.1) with the curated C2 gene set collection from the Molecular Signatures Database to identify pathways associated with high or low *OTUB2* expression. Results were reported as normalized enrichment scores, with statistical significance defined by a false discovery rate (FDR) < 0.25 and *p* < 0.05 [21].

### Cell culture

Human TNBC cell lines (MDA-MB-231, BT-549, Hs578T, and MDA-MB-468) and HEK293T cells (human embryonic kidney cell line) were cultured in Dulbecco’s Modified Eagle’s Medium (DMEM) (Thermo Fisher Scientific, C11995500BT, Lenexa, KS, USA) supplemented with 10% fetal bovine serum (FBS) (Excell Bio, FSP500, Suzhou, China) and antibiotics (100 U/mL penicillin and 100 μg/mL streptomycin) (Beyotime, C0222, Shanghai, China). MDA-MB-157 cells were cultured in Leibovitz’s L-15 medium (Thermo Fisher Scientific, 11415064, USA) supplemented with 10% FBS and antibiotics. Human normal mammary epithelial cells (MCF10A) were cultured in DMEM/F12 medium (Thermo Fisher Scientific, C11330500BT, USA) supplemented with 10% FBS and antibiotics [22,23]. All cells were incubated at 37°C in a humidified incubator with 5% CO_2_ atmosphere. Mycoplasma contamination was regularly monitored and tested.

### Plasmid construction and transfection

The OTUB2 siRNA (siOTUB2) constructs were synthesized by GenePharma (Suzhou, China), and the transfection control sequence (siNC) was purchased from GenePharma (A06001). The OTUB2 (NM_023112.4) and TRAF6 (NM_145803.3) coding regions were tagged with MYC and FLAG, respectively, and cloned into empty loading plasmids to obtain the overexpression plasmids pSin-EF2-puro-OTUB2-MYC and pSin-EF2-TRAF6-FLAG. The shRNA sequences targeting OTUB2 were synthesized and inserted into the vector pLKO.1-RFP to obtain the pLKO.1-shOTUB2#1/2 plasmids. Stable cell lines with OTUB2 overexpression or knockdown were generated via infection with retrovirus packaged from HEK293T cells. The viral supernatants were infected into target cells for 48 h, and then puromycin (1 μg/mL) was used to select cell lines for 1 week [24]. The target sequences of siRNA and shRNA are listed in Table 1.

**Table 1:** The primer sequence used in this study

Name	Sequences (5′–3′)
**Primers for plasmid constructs (Vector plasmid: pSIN-EF2-puro)**	
OTUB2-F	CGGAATTCGCCACCATGAGTGAAACATCTTTCAA
OTUB2 Flag-Tag R	GGACTAGTTCACTTATCGTCGTCATCCTTGTAATCATGTTTATCGGCTGCATAAA
OTUB2 Myc-Tag R	GGACTAGTTCACAGATCCTCTTCAGAGATGAGTTTCTGCTCATGTTTATCGGCTGCATAAAG
TRAF6-F	CGGAATTCATGAGTCTGCTAAACTGTGA
TRAF6 Flag-Tag R	GGACTAGTCTACTTGTCATCGTCGTCCTTGTAATCTACCCCTGCATCAGTACTTC
TRAF6 Myc-Tag R	GGACTAGTCTACAGATCCTCTTCAGAGATGAGTTTCTGCTCTACCCCTGCATCAGTACTTC
**RT-qPCR primers**	
OTUB2-Human-F	GCTGGCTTTGAGGAGCACAAGT
OTUB2-Human-R	CTGGTCGTTGAACACCTTCAGC
GAPDH-Human-F	GTCTCCTCTGACTTCAACAGCG
GAPDH-Human-R	ACCACCCTGTTGCTGTAGCCAA
TRAF6-Human-F	CAATGCCAGCGTCCCTTCCAAA
TRAF6-Human-R	CCAAAGGACAGTTCTGGTCATGG
**siRNA sequences**	
siNC	UUCUCCGAACGUGUCACGUTT
siOTUB2#1	ATCTTTCAACCTAATATCAGAAA
siOTUB2#2	TTCAACCTAATATCAGAAAAATG
**shRNA sequences (Vector plasmid: pLKO.1-RFP)**	
shNC	ATGGACTATCATATGCTTACCGTA
shOTUB2#1	CCTTCCGTTTACCTGCTCTAT
shOTUB2#2	CGAGATGGATACCGCCCTGAA

MDA-MB-231 and BT-549 cells were transfected with the indicated siRNAs or plasmids using Lipofectamine 3000 (Invitrogen, L3000015, Lenexa, KS, USA) or Neofect (Neofect Biotechnologies, TF20121201, Beijing, China) according to the manufacturer’s instruction. The transfection efficiency was evaluated by quantitative RT-qPCR and western blotting after 24–48 h of transfection.

### RT-qPCR assay

Total RNA was extracted by RNA Quick Purification kit (ESscience, RN001, Shanghai, China), and cDNA was synthesized using the NoScript Reverse Transcription System (Promega, A5001, Beijing, China). Quantitative PCR was performed with ChamQ SYBR qPCR Master Mix (Vazyme, Q311-02, Nanjing, China) on a LightCycler 480 II System (Roche Molecular Diagnostics, 5015243001, USA). The RT-qPCR cycling conditions were as follows: initial denaturation at 95°C for 30 s, followed by 40 cycles of denaturation at 95°C for 10 s, and annealing at 60°C for 30 s. A melting curve analysis was conducted using the instrument’s default settings. *GAPDH* was used as an internal control, and relative expression was calculated by the 2^−ΔΔC^_T_ method [25]. The primer sequences are shown in Table 1.

### Cell viability assay

MDA-MB-231 and BT-549 cells were plated into 96-well plates at a density of 800 cells per well. On the indicated day (days 0, 1, 2, 3, and 4), 10 μL/well of Cell Counting Kit-8 (CCK-8) reagent (TargetMol, C0005, Shanghai, China) was added to the 96-well plates [26]. After incubation at 37°C for 2 h, the absorbance of each well at 450 nm was detected on a spectrophotometer (EPOCH2, BioTek Instruments, Inc., Winooski, VT, USA).

### Colony formation assay

Single-cell suspensions (800–1000 cells per well) were seeded in 6-well plates. Two weeks later, the colonies were rinsed with PBS, fixed with methanol, and stained with crystal violet (Beyotime, C0121, Shanghai, China). The number of colonies was then counted.

### Wound-healing assay

MDA-MB-231 and BT-549 cells were seeded in 12-well plates and cultured to near confluence. A wound was created in the cell monolayer using a sterile 10 μL pipette tip. Images of the wound area were captured at specific time points (0 and 48 h) using an inverted microscope (Nikon ECLIPS Ti2-U, Nikon Corporation, Tokyo, Japan).

### Transwell assay

The migration assay was conducted using Transwell Chambers (LABSELECT, 14341, Hefei, China). MDA-MB-231 or BT-549 cells (1 × 10^5^ cells per well) were suspended in 200 μL serum-free medium and added into the upper chamber. The lower chamber was filled with 500 μL medium supplemented with 10% FBS. After incubation for 12 or 16 h, cells were fixed with methanol, stained with 0.1% crystal violet, and captured by an inverted microscope (NIKON Eclipse Ti2).

### Western blot assay

Cell lysis was performed on ice using RIPA buffer (Merck Millipore, R0278, USA) with a protease inhibitor cocktail (Beyotime, P1046, China). The lysate proteins were isolated using 7.5%–15% SDS-PAGE gels (Epizyme, PG111-3, Shanghai, China) and subsequently transferred to Polyvinylidene Fluoride (PVDF) membranes (Merck Millipore, IPVH00010, Burlington, MA, USA). Following blocking of the membranes with 5% nonfat milk, primary antibodies against various proteins, including OTUB2 (1:1000, Affinity, AF9147, Liyang, China), Tubulin (1:5000, Proteintech, 11224-1-AP, Wuhan, China), DYKDDDDK-Tag (1:2000, Proteintech, 66008-4-Ig), Myc-Tag (1:1000, CST, 2278S, USA), HA-Tag (1:1000, Sigma, H9658, Burbank, CA, USA), AKT (1:2000, Proteintech, 10176-2-AP), Phospho-AKT (1:1000, CST, 4060S), were applied. After washing 3 times with TBST (TBS: Biosharp, BL602A-25, Hefei, China; Tween-20: Solarbio, T8220, Beijing, China), the membranes were incubated with specific secondary antibodies (1:5000, Proteintech, SA00001-2-100UL, SA00001-1-100UL) at room temperature for 1 h. The target protein bands were visualized using an enhanced chemiluminescence substrate (APPLYGEN, P1050-500, Beijing, China) and imaged with the ChemiDic MP Imaging System (Bio-Rad, 12003154, Boulder, CO, USA) [24]. The relative protein expression was quantified using ImageJ (version 1.52a, NIH, Bethesda, MD, USA) software by measuring band intensities. To account for variations in sample loading, the grayscale values of the target protein were normalized to Tubulin. The normalized expression levels of the experimental groups were then compared to the control group, with the control set as the baseline, to determine the relative protein expression [27].

### Co-immunoprecipitation (Co-IP)

Cells were lysed in IP lysis buffer (Beyotime, P0013), scraped off from the petri dish by a cell scraper, and transferred to a tube for centrifugating and sonicating. The protein supernatant was incubated with anti-Flag magnetic beads (Selleck, B26102, Houston, TX, USA) overnight at 4°C. The beads were washed with IP wash buffer and boiled in 1× SDS loading buffer for WB analysis [28].

### Ubiquitin assay

HEK293T cells were transfected for 24 h and then incubated with 10 μM MG132 (Selleck, S2619, USA), a proteasome inhibitor, for 6 h. Subsequently, cells were lysed in RIPA buffer containing 1% SDS on ice, and the lysates were heated at 100°C for 5 min to denature the proteins. The denatured lysates were then diluted to 0.1% SDS concentration with RIPA buffer and immunoprecipitated with Anti-Flag beads (Selleck, B26102, USA). The level of ubiquitination was detected using western blotting with an anti-HA-Tag antibody (1:1000, Sigma, H9658).

### Immunohistochemistry (IHC)

Immunohistochemistry was performed as previously described [28]. Six pairs of matched cancerous and adjacent normal tissue samples from patients with TNBC were obtained from Sun Yat-Sen University Cancer Center (G2023-213-01). In brief, the sections were sequentially deparaffinized and rehydrated with xylene and ethanol, and endogenous peroxidase activity was blocked. The sections were then subjected to antigen retrieval. Finally, the sections were incubated with anti-OTUB2 antibody (1:200, Affinity, AF9147) overnight at 4°C, followed by the addition of secondary antibody (ZSGB-BIO, PV-6000, Beijing, China) and incubation at room temperature for 25 min, DAB (ZSGB-BIO, ZLI-9018) development and hematoxylin (Beyotime, C0107) staining. The stained slides were scanned and imaged using a Jiangfeng automated slide scanning system (KF-PRO-020, China) evaluated by an experienced pathologist blinded to the clinical data.

### Animal experiments

Female BALB/c nude mice, aged 3–4 weeks, were obtained from Charles River Laboratories (Guangzhou, China), housed in a specific pathogen-free (SPF) facility under controlled conditions (22°C–24°C, 12/12-h light/dark cycle) with autoclaved food and water ad libitum. All animal procedures were approved by the Animal Ethics Committee of Sun Yat-Sen University Cancer Center (L025501202404001). The mice were randomly assigned to two groups. To assess tumor growth, 1 × 10^7^ shOTUB2#1 or control vector MDA-MB-231 cells were resuspended in 200 μL of PBS and injected into the mammary fat pads of the mice (n = 5 per group) [23]. Tumor volume was measured every three days starting from day 6 post-injection (Volume = (length × width × width)/2). After 18 days, the mice were sacrificed, and the tumors were isolated for weighing and photographing.

### Statistics and reproducibility

Data are presented as the mean ± standard deviation (SD) from three independent experiments performed in duplicate. Continuous variables were compared using Student’s *t*-test and two-way ANOVA followed Dunnett’s multiple comparisons test. Statistical analyses were performed using GraphPad Prism software (version 8.0.1, GraphPad Software, LLC), and *p*-value < 0.05 was considered statistically significant.

## Results

### The Expression of OTUB2 was increased in TNBC

**Figure 1:**
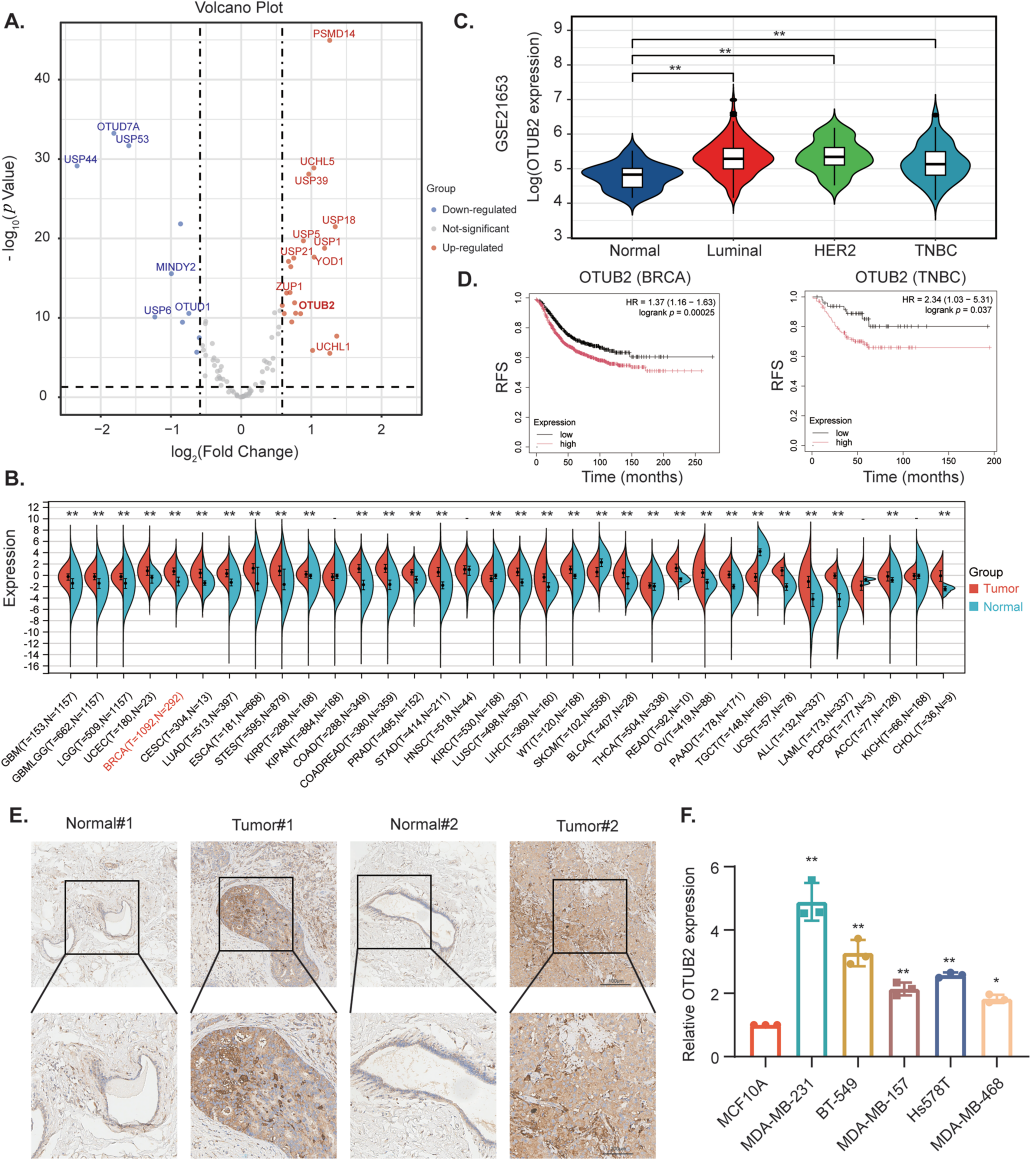
The expression of OTUB2 was significantly increased in TNBC. (A) Volcano plot showed the differentially expressed genes of deubiquitinases in TNBC based on TCGA datasets; (B) The expression of *OTUB2* in pan-cancer based on TCGA cancer and normal data analyzed; (C) The expression of *OTUB2* in four subtypes of breast cancer; (D) The prognosis induced by *OTUB2* expression was analyzed by Kaplan-Meier in patients with BRCA or TNBC; (E) Immunohistochemistry exhibited OTUB2 expression in tumor tissues was higher than normal; (F) Relative mRNA levels of *OTUB2* in TNBC cell lines (MDA-MB-231, BT-549, MDA-MB-157, Hs578T, MDA-MB-468) and breast epithelial cell line MCF10A (*p < 0.05, **p < 0.01).

We first analyzed the expression of deubiquitinases (DUBs) in TNBC using the TCGA-BRCA dataset, which was downloaded from the UCSC Xena platform. From the dataset, we specifically selected TNBC samples based on clinical information. Then, we conducted a differential analysis on 91 DUBs retrieved from the DUBase database (https://ehubio.ehu.eus/dubase/) (accessed on 02 March 2025). The results showed that 10 DUBs were downregulated, while 21 were upregulated in TNBC. Among these, OTUB2, a DUB with limited previous research in TNBC, exhibited high expression, which prompted subsequent investigation (Fig. 1A). To further explore the significance of OTUB2 in cancer, we examined its expression across various cancer types (http://www.sangerbox.com/) (accessed on 02 March 2025). Compared with normal tissues, the expression of *OTUB2* was generally upregulated in solid tumors (Fig. 1B). We further validated *OTUB2* expression in pan-cancers using the UALCAN (https://ualcan.path.uab.edu/) (accessed on 02 March 2025) and TIMER2.0 (http://timer.cistrome.org/) (accessed on 02 March 2025) databases (Fig. S1A,B). Additionally, analysis of the GSE21653 dataset revealed that *OTUB2* expression was significantly higher in TNBC tissues compared to normal tissues (Fig. 1C). The GEPIA (http://gepia2.cancer-pku.cn/) (accessed on 02 March 2025) and UALCAN databases also confirmed that the transcriptional level of *OTUB2* was elevated in breast cancer tissues (Fig. S1C,D). Kaplan-Meier plots (https://kmplot.com/analysis/) (accessed on 02 March 2025) showed that patients with higher OTUB2 expression in breast cancer or TNBC had shorter relapse-free survival (RFS) (Fig. 1D), suggesting that OTUB2 may influence the prognosis of TNBC patients. Immunohistochemical (IHC) staining revealed that OTUB2 was predominantly localized in the cytoplasm and exhibited stronger staining in TNBC tissues compared to adjacent normal tissues (Fig. 1E). Moreover, qPCR analysis of mRNA extracted from TNBC cell lines showed that *OTUB2* expression was significantly higher in TNBC cell lines compared to normal breast epithelial cells (Fig. 1F).

### OTUB2 promotes TNBC cell proliferation in vitro and tumor growth

**Figure 2:**
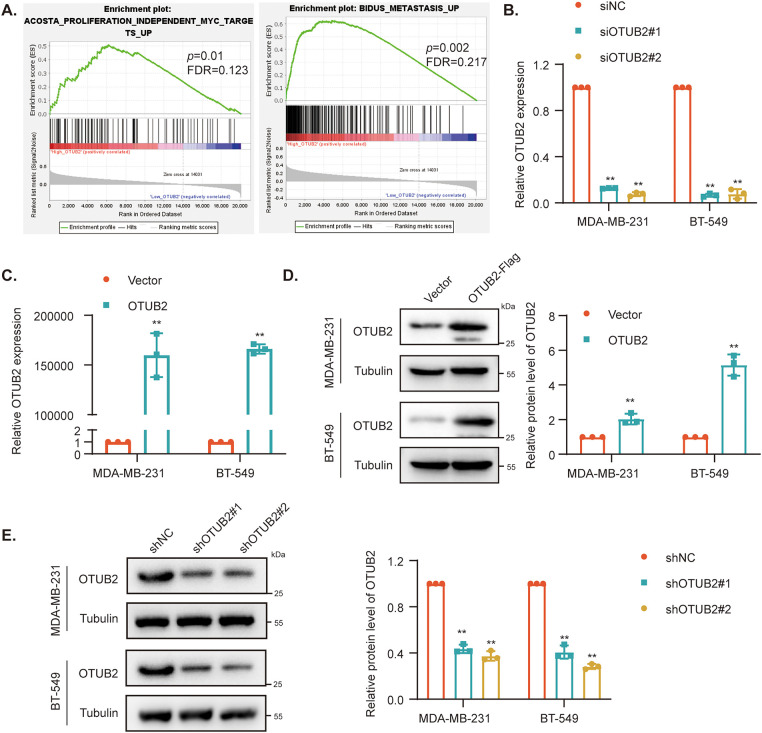
OTUB2 expression was associated with proliferation and metastasis. (A) In GSEA analysis using the TCGA database, the gene sets associated with proliferation and metastasis were significantly enriched in TNBC samples with high *OTUB2* expression; (B, C) qPCR analysis of OTUB2 mRNA expression following the indicated transfection; (D, E) Western Blotting showing the protein expression of OTUB2 in stable MDA-MB-231 and BT-549 cells (**p < 0.01).

**Figure 3:**
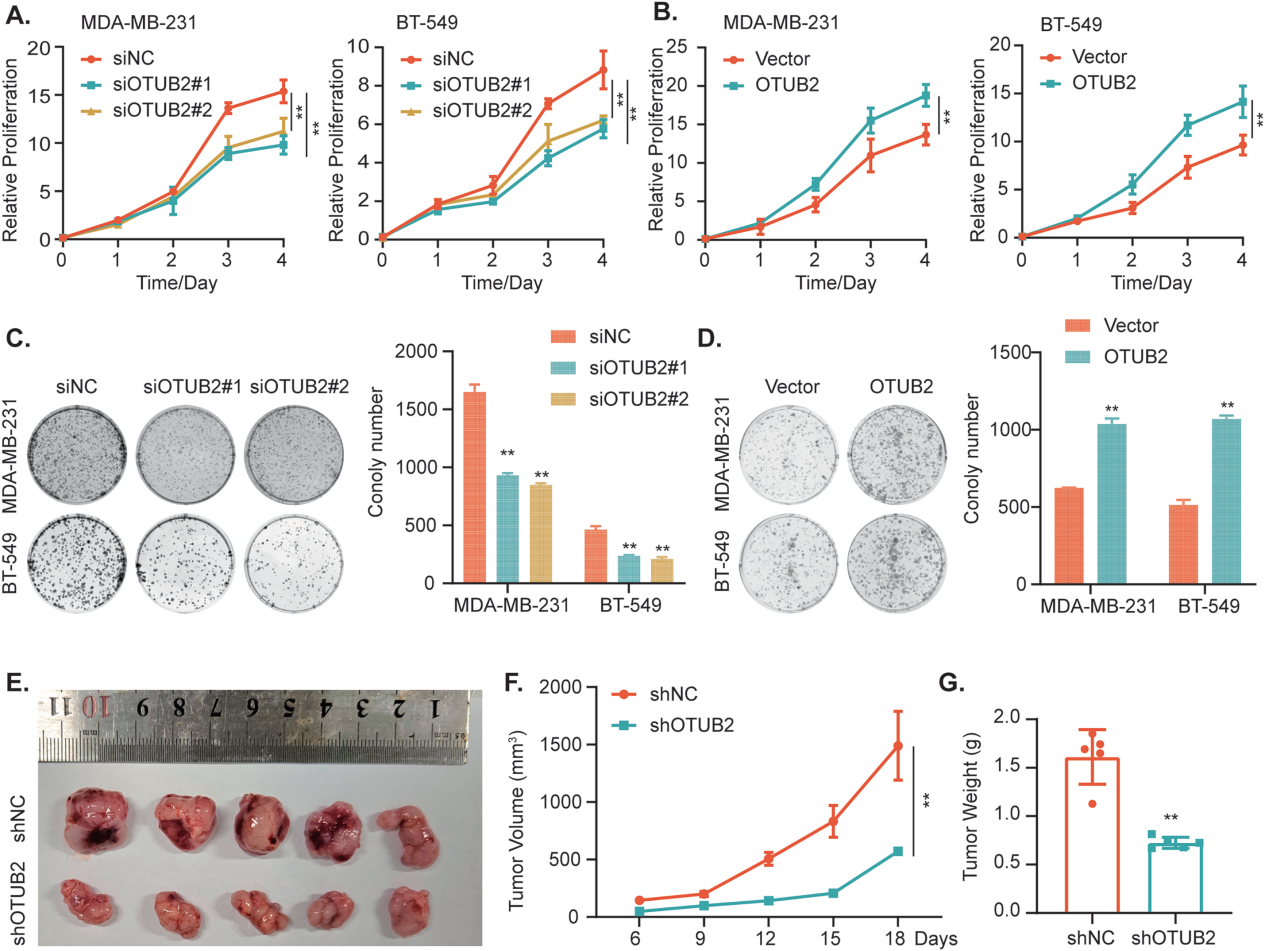
OTUB2 promoted TNBC cell proliferation *in vitro* and tumor growth. (A, B) The effect of OTUB2 on cell proliferation was measured by a CCK8 assay. (C, D) The colony formation assay was used to measure cell proliferation. Representative picture (left) and quantitative statistics (right) of colony numbers. (E–G) Influence of OTUB2 on the growth of MDA-MB-231 cells-derived tumors in mice. Tumor images, growth curves, and tumor weights are shown (**p < 0.01).

To further investigate the potential biological functions of OTUB2, we conducted gene set enrichment analysis (GSEA), which revealed that *OTUB2* was significantly associated with processes such as proliferation and metastasis (Fig. 2A). We then established stable OTUB2 overexpression or knockdown cell lines in MDA-MB-231 and BT-549 cells, with efficiency confirmed by qPCR and Western blotting (Fig. 2B–E). To assess the effect of OTUB2 on the proliferation ability of TNBC cells, we transiently overexpressed or knocked down the expression of OTUB2 in MDA-MB-231 and BT-549 cells and performed cell viability assays and clonogenic assays. The results demonstrated that OTUB2 knockdown significantly inhibited TNBC cell growth, while overexpression promoted cell growth in a time-dependent manner (Fig. 3A,B). The clonogenic assay further confirmed these findings, showing a similar trend in long-term proliferation (Fig. 3C,D). Finally, we injected MDA-MB-231 cells with stable shOTUB2#1 into the fat pads of female nude mice. The subcutaneous xenograft model showed that OTUB2 knockdown suppressed tumor growth *in vivo* (Fig. 3E–G).

### OTUB2 promotes migration of TNBC cells

**Figure 4:**
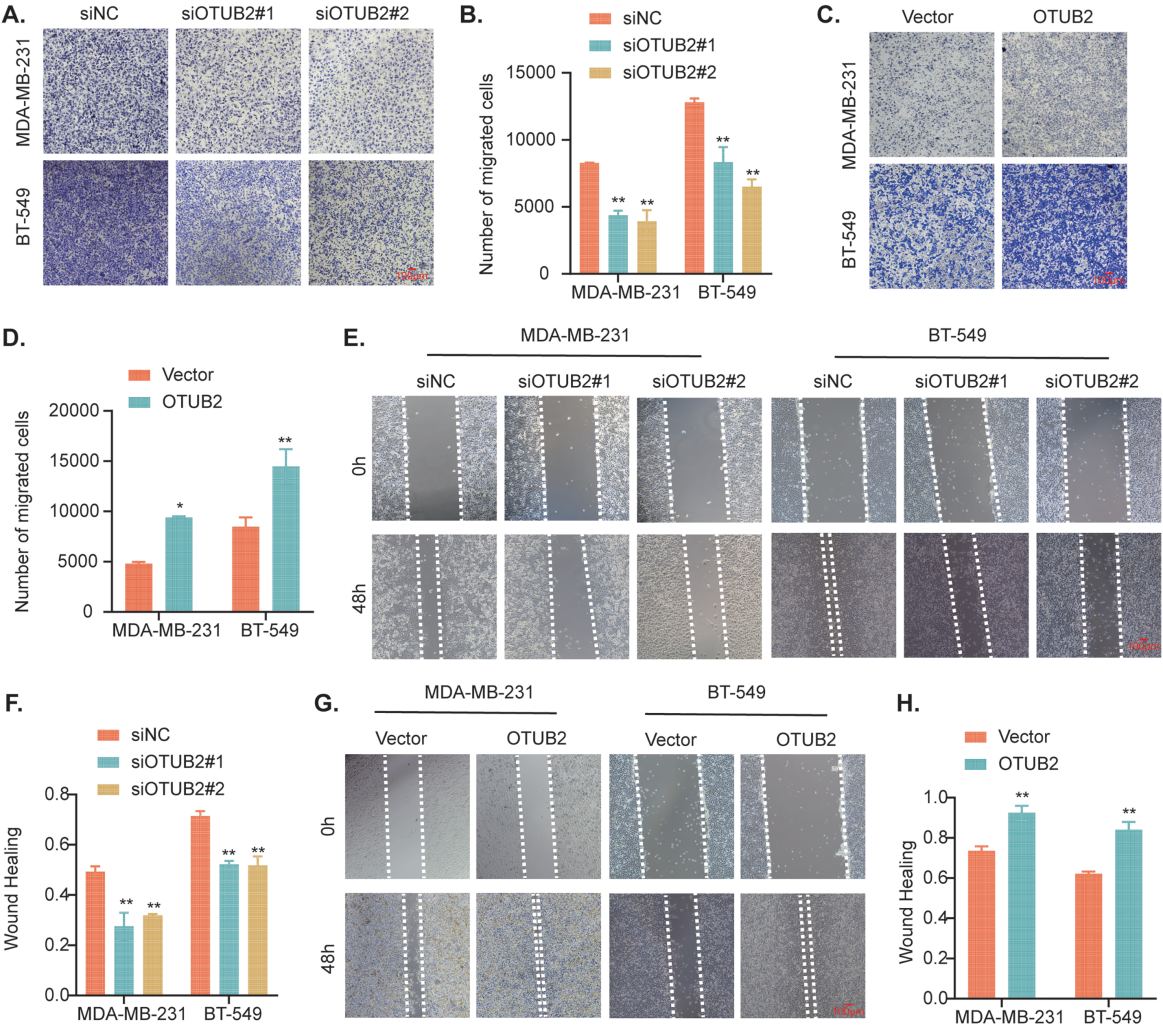
OTUB2 promoted migration of TNBC cells. (A, B) The migratory abilities of MDA-MB-231 and BT-549 cells transfected with siOTUB2 determined by Transwell assay; (C, D) Transfected with Flag-OTUB2 plasmid or its empty vector; (E, F) The migratory abilities of MDA-MB-231 and BT-549 cells transfected with siOTUB2 determined by wound healing assay; (G, H) Transfected with Flag-OTUB2 plasmid or its empty vector (*p < 0.05, **p < 0.01).

The results of the Transwell assay showed that the knockdown of OTUB2 significantly impaired the migratory and invasive abilities of TNBC cells (Fig. 4A,B), indicated by the reduced number of cells migrating through the membrane. In contrast, overexpression of OTUB2 enhanced the migratory abilities of MDA-MB-231 and BT-549 cells (Fig. 4C,D). These findings were further corroborated by the wound-healing assay, which assessed the ability of cells to close a scratch wound. In this assay, OTUB2 overexpression significantly accelerated the wound closure rate, indicating enhanced cell migration (Fig. 4G,H). Conversely, the knockdown of OTUB2 resulted in slower wound healing, reinforcing the Transwell results and confirming that OTUB2 depletion inhibits TNBC cell migration (Fig. 4E,F). Taken together, these complementary assays provide strong evidence that OTUB2 plays a critical role in enhancing the migratory and invasive abilities of TNBC cells, which are key processes in tumor migration.

### OTUB2 deubiquitinates TRAF6 and activates AKT

**Figure 5:**
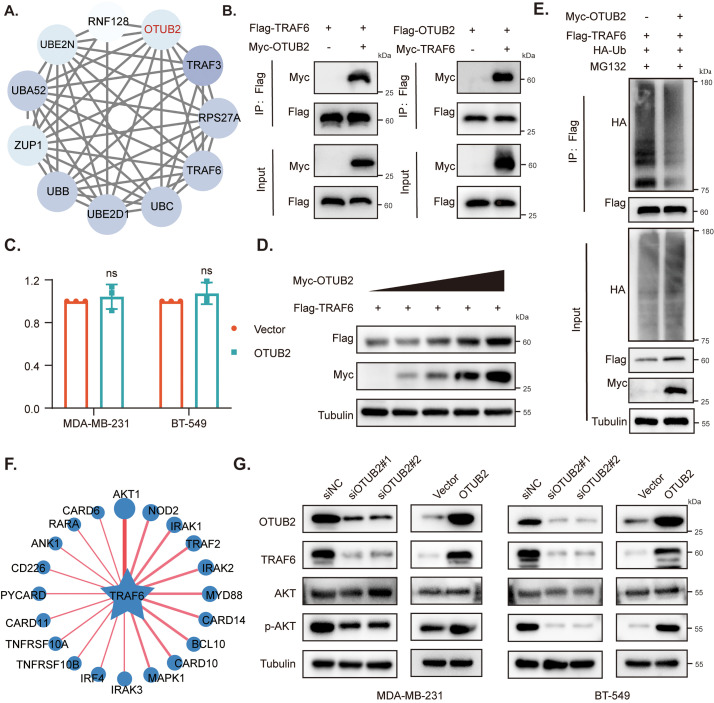
OTUB2 deubiquitinated TRAF6 and activated AKT. (A) Bioinformatics analysis of interaction molecules of OTUB2; (B) Co-IP with anti-Flag antibody showing interactions between exogenous OTUB2 and TRAF6 in HEK293T cells; (C) qPCR analysis was performed to assess the mRNA levels of TRAF6 in MDA-MB-231 and BT-549 cells following OTUB2 overexpression; (D) OTUB2 promoted TRAF6 protein expression in a dose-dependent manner; (E) HEK293T cells transfected with Flag-TRAF6, HA-Ub and Myc-OTUB2 or the empty plasmids following MG132 treatment (10 μm, 6 h) were subjected to denatured-IP and immunoblotted with the indicated antibodies; (F) UbiBrowser 2.0 was used to predict substrates of E3 ligase; (G) Western blot analysis was performed to assess AKT, p-AKT, and TRAF6 protein expression levels in OTUB2 knockdown MDA-MB-231 and BT-549 cells (ns: *p* > 0.05).

To further investigate the mechanism by which OTUB2 regulates proliferation and migration, we analyzed its potential interacting partners using the STRING online tool. This analysis revealed a potential interaction between OTUB2 and TRAF6 (Fig. 5A), and subsequent studies confirmed that TRAF6 is a substrate of OTUB2. Numerous studies have confirmed that TRAF6 is closely associated with tumorigenesis and is a potential target in malignant tumors. For instance, POU5F1 promotes gastric cancer (GC) cell proliferation and migration by inhibiting TRAF6 ubiquitination and degradation, thereby activating the NF-κB pathway [29], and KDM48 stimulates TRAF6-mediated AKT activation and facilitates the progression of rectal cancer [30]. Therewith, we demonstrated the exogenous binding between OTUB2 and TRAF6 in HEK293T cells by Co-immunoprecipitation (Co-IP) assays (Fig. 5B). Furthermore, we observed that overexpression of OTUB2 increased the protein levels of TRAF6 in a dose-dependent manner, although it did not affect TRAF6 mRNA expression (Fig. 5C,D, Fig. S2A), indicating that OTUB2 regulates TRAF6 expression at the post-transcriptional level. OTUB2, as a member of the deubiquitinase family, promotes substrate stability by cleaving ubiquitin chains and exhibits ubiquitin hydrolase activity. Therefore, we examined the effects of OTUB2 on TRAF6 ubiquitination and found that OTUB2 overexpression reduced the polyubiquitination of TRAF6 (Fig. 5E).

Given that TRAF6 functions as an E3 ubiquitin ligase with a RING domain, we utilized the UbiBrowser 2.0 (http://ubibrowser.bio-it.cn/) (accessed on 02 March 2025) to identify its protein substrates. Among the predicted substrates, AKT, which had the highest score, drew our attention (Fig. 5F). TRAF6 has been identified as a direct E3 ligase for AKT, and its ubiquitination is a crucial step in oncogenic AKT activation [31]. Based on these findings, we hypothesized that OTUB2 activates AKT by deubiquitinating TRAF6, thereby exerting a pro-oncogenic effect. To test this hypothesis, we transiently knocked down OTUB2 expression, and Western blot analysis revealed a decrease in AKT phosphorylation. Conversely, overexpression of OTUB2 led to a corresponding increase in p-AKT levels (Fig. 5G, Fig. S2B–E).

## Discussion

Breast cancer is the most prevalent women malignant tumor, posing a significant threat to human health. TNBC is considered the most aggressive subtype of breast cancer due to its rapid progression, high metastatic potential, and resistance to standard treatments. In recent years, an increasing number of studies have reported potential therapeutic targets for TNBC [32,33]. However, there is still a lack of new molecular targets and effective drugs against these new targets.

The ubiquitin-proteasome system (UPS) is a critical protein degradation pathway in eukaryotic cells. It consists of Ub-activating (E1), Ub-conjugating (E2), Ub-ligating (E3), DUBs, and proteasomes [34]. The UPS plays a crucial role in protein degradation and the regulation of basic cellular processes, making its components promising targets for anticancer therapies. DUB inhibitors function by directly interacting with the catalytic site, thereby modulating the target DUB into either an inactive or active conformation. In recent years, potent and selective small-molecule DUB inhibitors have been developed, showing significant potential as novel therapeutic agents for various diseases [35].

OTUB2, a cysteine protease with deubiquitinating enzyme activity, has garnered increasing attention for its effects on malignant tumor progression. Studies have reported that OTUB2 facilitates cancer metastasis via activating YAP and TAZ [36] and enhances the proliferation of gastric cancer cells by deubiquitinating KRT80 [37]. Additionally, Xu et al. also demonstrated that OTUB2 promotes colorectal cancer growth by regulating β-catenin signaling [38]. Although previous studies have illuminated the role of OTUB2 in tumor progression, its relationship with proliferation and metastasis in triple-negative breast cancer is still unclear. Here, we showed that OTUB2 expression was significantly upregulated in TNBC tissue samples compared with normal tissues and was correlated with patients’ poor prognosis. Furthermore, overexpression of OTUB2 promoted TNBC cell proliferation and migration, whereas silencing of OTUB2 resulted in the opposite effect. The findings suggest that OTUB2 may act as a tumor promoter in the carcinogenesis of TNBC.

There are seven members of the tumor necrosis factor receptor-related factors (TRAFs) family [39], among which TRAF6 is up-regulated in various malignant tumors and closely related to tumorigenesis and progression [40]. Starczynowski et al. reported that TRAF6 is one of the amplified candidate oncogenes on chromosome 11p13, inhibition of TRAF6 reduced NF-κB activation and suppressed tumor growth of human lung cancer [41]. Besides, TRAF6 also activates phosphoinositide 3-kinase (PI3K) and mitogen-activated protein kinase (MAPK) directly [42]. Multiple studies have demonstrated that TRAF6 participates in a variety of signaling pathways and regulates tumor cell proliferation, metastasis, immune response, and survival. Protein-protein interaction network analysis (PPI) suggested that TRAF6 could be a substrate of OTUB2 [43]. The experimental results further confirmed that OTUB2 reduced the ubiquitination of TRAF6, thereby positively regulating its protein expression.

Previous studies have shown that AKT signaling plays a pivotal role in various biological processes, including cell proliferation, invasion and apoptosis [44]. The excessive activation of the PI3K/AKT oncogenic signaling pathway is a frequent event in human tumors [45,46]. Dysregulation of this pathway is commonly observed in TNBC and is associated with poor prognosis and resistance to treatment [47,48]. In our current study, TRAF6 and phosphorylated AKT expression were repressed after OTUB2 knockdown, which suggested that AKT signaling might be associated with the regulation of cell proliferation and migration mediated by OTUB2 in TNBC.

The approval of bortezomib for treating multiple myeloma has confirmed the proteasomes as a valid target for anti-cancer therapy [49]. Regarding DUBs, pimozide, a USP1 inhibitor, has shown the ability to reduce glioblastoma (GBM) growth in xenograft models and has entered Phase I/II clinical trials for GBM [50]. USP7 inhibitors such as p5091, p220077, and p50429 enhance the ubiquitination and degradation of murine double minute 2 (MDM2), thereby inducing apoptosis in bortezomib-resistant multiple myeloma (MM) cells. These compounds are currently undergoing preclinical trials [51,52]. Unfortunately, no inhibitors targeting OTUB2 have yet been successfully developed. Based on preclinical evidence and proof-of-concept data, Ren et al. proposed that targeting OTUB2 with a specific inhibitor, OTUB2-IN-1 is a promising cancer treatment strategy [53]. Therefore, OTUB2-targeted inhibitors hold the potential for development as stand-alone therapies or be used to enhance therapeutic effects in the near future.

However, we primarily focused on the role of OTUB2 in TNBC without investigating its expression and functional significance in other breast cancer subtypes. Given the molecular heterogeneity of breast cancer, it remains unclear whether OTUB2 exerts similar oncogenic effects in hormone receptor-positive or HER2-positive breast cancer. Additionally, while our findings suggest that OTUB2 promotes TNBC cell migration, this conclusion is based solely on *in vitro* assays, and we lack *in vivo* metastasis models to further validate its role in tumor dissemination. Establishing appropriate *in vivo* models, such as orthotopic or tail vein injection metastasis models, will be essential to confirm whether OTUB2 truly facilitates metastasis in a physiological setting. Addressing these limitations in future studies will provide a more comprehensive understanding of OTUB2’s function in breast cancer and its potential as a therapeutic target.

## Conclusions

**Figure 6:**
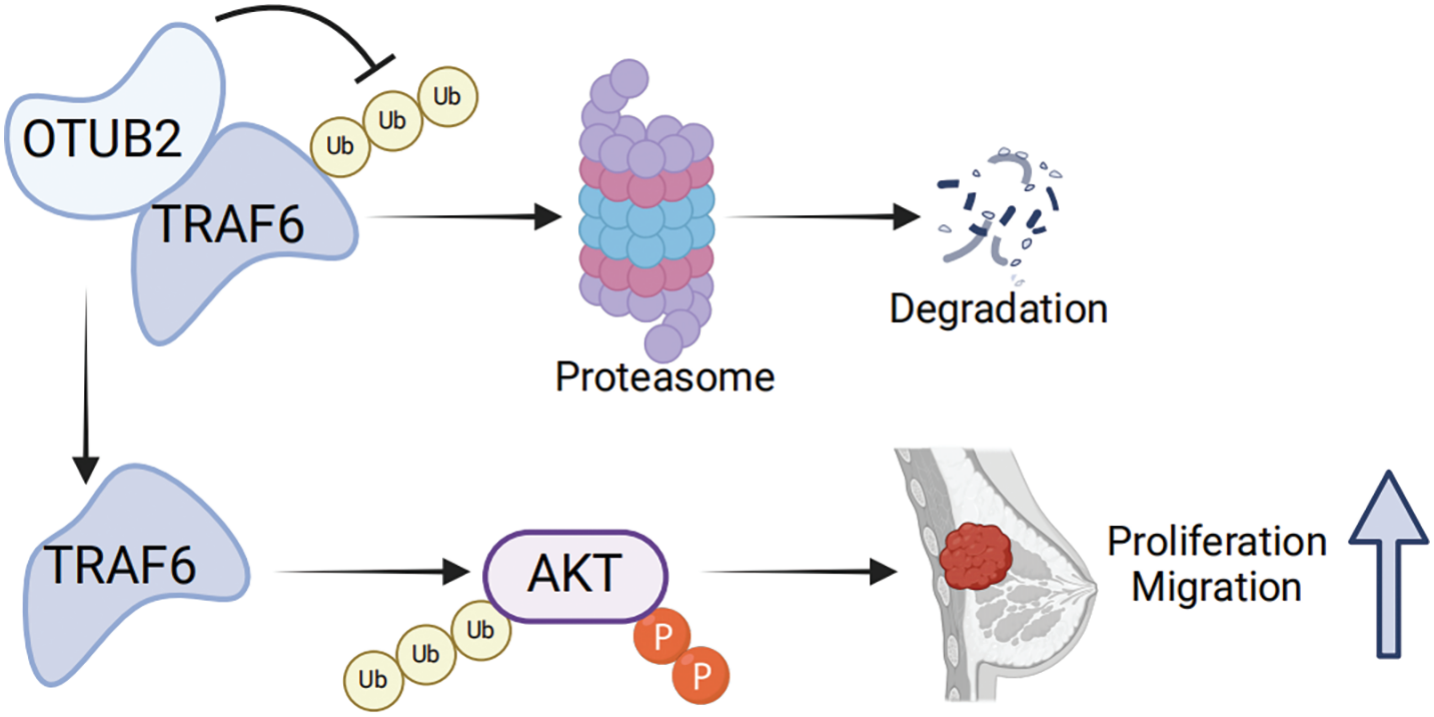
Proposed working model. The figure was generated using Biorender.com. OTUB2 inhibits the degradation of TRAF6 protein in a ubiquitin-proteasome pathway, promotes TNBC proliferation and migration.

In conclusion, our experiments confirmed that OTUB2 is overexpressed in TNBC tissues and cell lines as well as associated with poor prognosis. We suspect that OTUB2 contributes to this by rescuing the ubiquitination degradation of TRAF6, thereby activating the AKT pathway (Fig. 6). Thus, OTUB2 plays an important role in TNBC progression and could potentially serve as a novel therapeutic target for TNBC.

## Supplementary Materials

Figure S1 *OTUB2* expression was upregulated in TNBC. (A, B) Comparative expression of *OTUB2* between pan-cancers and corresponding normal tissues in the UALCAN and TIMER2.0 database; (C, D) OTUB2 expression between breast cancer and normal tissue (*p ＜ 0.05, **p ＜ 0.01, ***p ＜ 0.001).

Figure S2 **Protein quantification analysis** (A) Quantification of Flag-TRAF6 expression levels, normalized to Tubulin. (B-E) Quantification of OTUB2, TRAF6, p-AKT protein expression levels (ns: *p* > 0.05, *p ＜ 0.05, **p ＜ 0.01, ***p ＜ 0.001).

## Data Availability

The dataset used and analyzed during the current study are available from the corresponding author on reasonable request.

## Abbreviations

TNBC: Triple-negative breast cancer
GSEA: Gene set enrichment analysis
CCK-8: Cell Counting Kit-8
IHC: Immunohistochemistry
TRAF6: Tumor Necrosis Factor Receptor-Associated Factor 6
AKT: Protein kinase B
ER: Estrogen receptor
PR: Progesterone receptor
HER2: Human epidermal growth factor receptor-2
DUB: Deubiquitinase
GC: Gastric cancer
Co-IP: Co-immunoprecipitation
UPS: Ubiquitin-proteasome system
TRAFs: The tumor necrosis factor receptor-related factors
PI3K: Phosphoinositide 3-kinase
MAPK: Mitogen-activated protein kinase
GBM: Glioblastoma
MM: Multiple myeloma
MDM2: Murine double minute 2

## Supplementary Materials

The supplementary material is available online at https://doi.org/10.32604/or.2025.062767.
