# Supplementary figures and images for: OTUB2 promotes proliferation and metastasis of triple-negative breast cancer by deubiquitinating TRAF6
Source: Oncol Res. 2025 Apr 18;33(5):1135–47. doi: 10.32604/or.2025.062767 (PMC12034018; doi:10.32604/or.2025.062767)

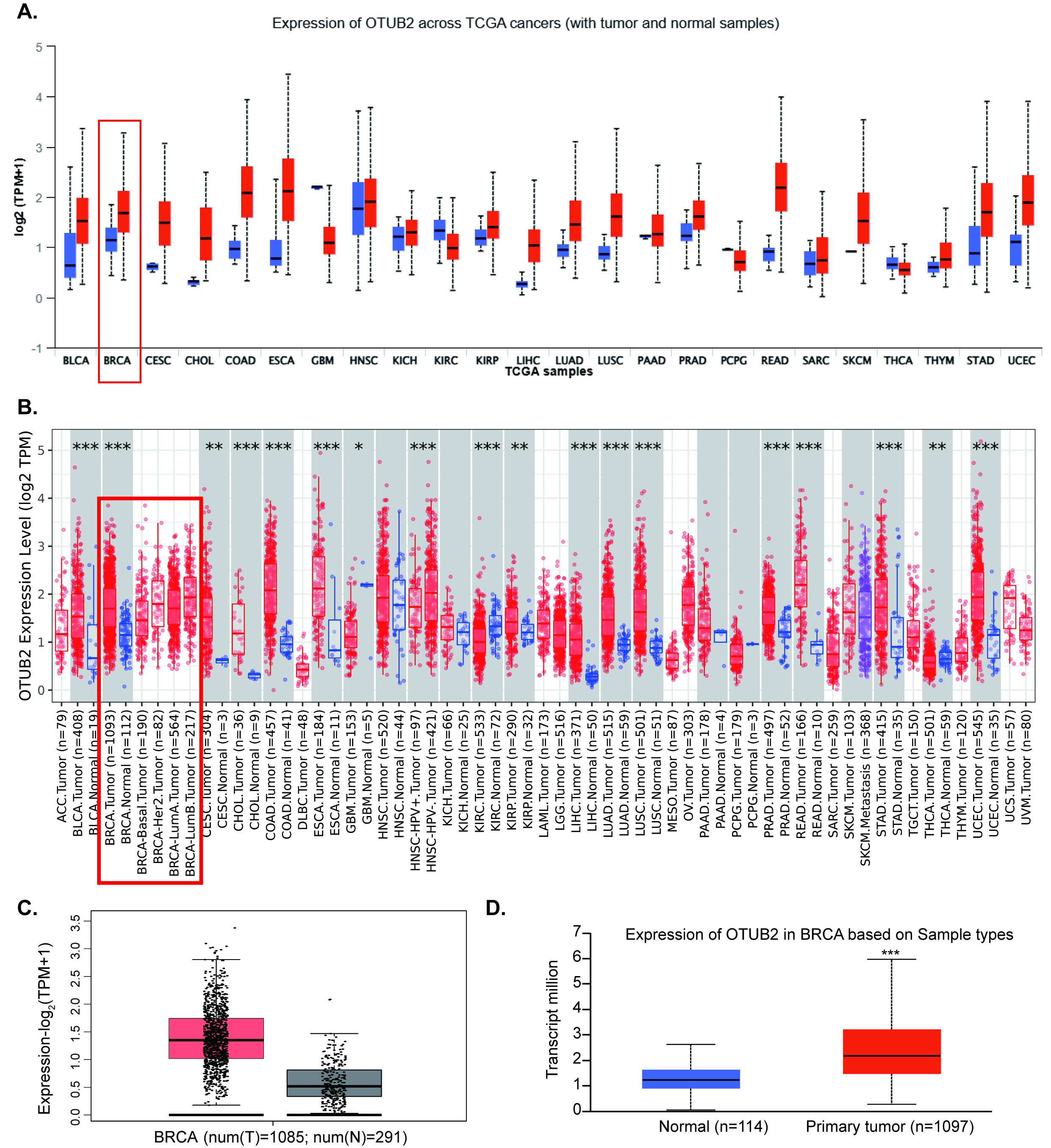

Supplement: Figure S1 [file OncolRes-33-62767-s001.tif]

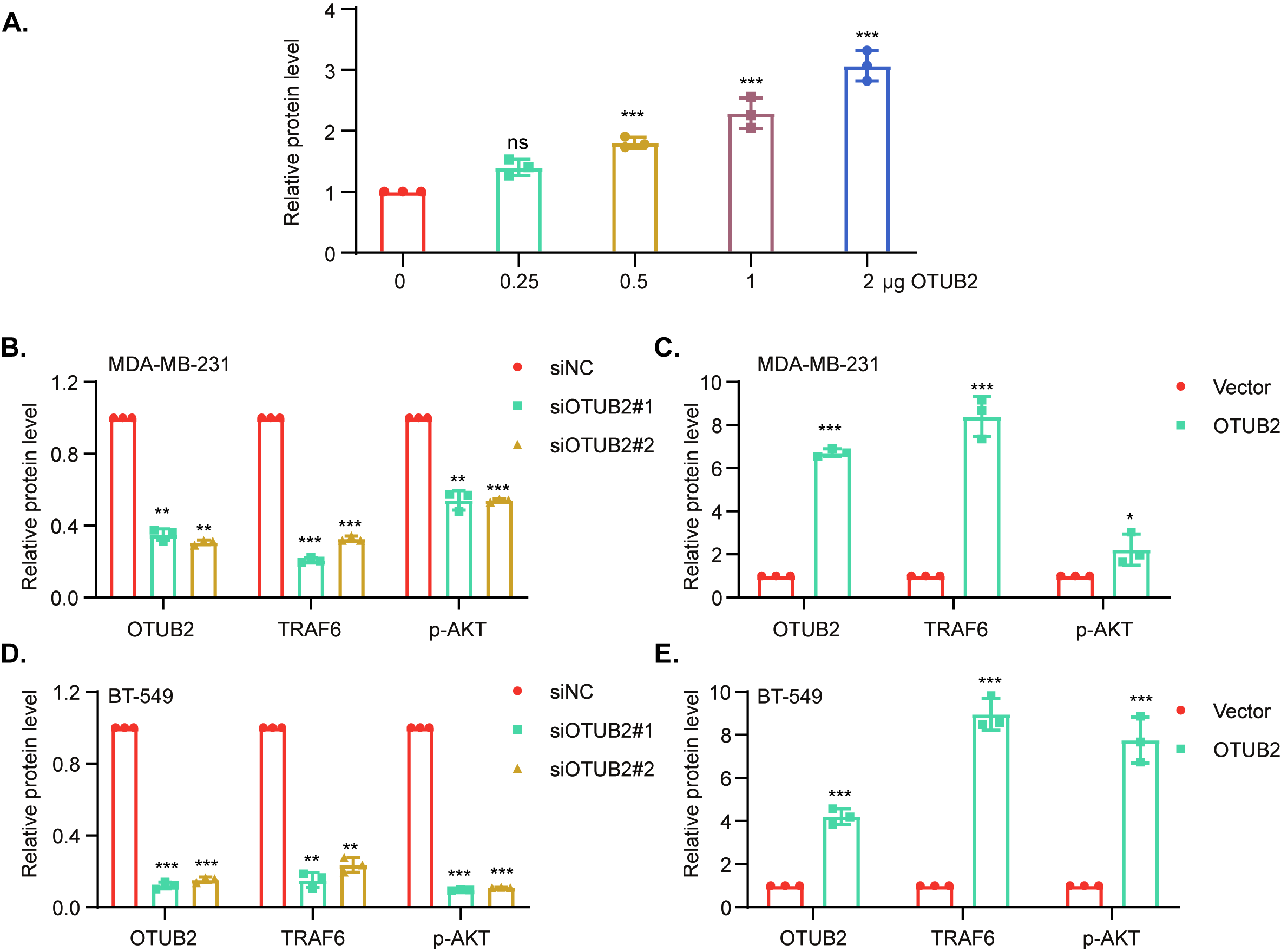

Supplement: Figure S2 [file OncolRes-33-62767-s002.tif]
